# Weight gain rate in the second and third trimesters and fetal growth in women with gestational diabetes mellitus: a retrospective cohort study

**DOI:** 10.1186/s12884-022-04762-4

**Published:** 2022-05-20

**Authors:** Miao Hong, Feng Liang, Zheng Zheng, Huimin Chen, Yi Guo, Kuanrong Li, Xihong Liu

**Affiliations:** 1 Clinical Data Center, Institute of Pediatrics, Guangzhou Women and Children’s Medical Center, Guangzhou Medical University, 9 Jinsui Road, Guangzhou, 510623 Guangdong China; 2 Department of Obstetrics, Guangzhou Women and Children’s Medical Center, Guangzhou Medical University, Guangzhou, 510623 Guangdong China; 3 Department of Clinical Nutrition, Guangzhou Women and Children’s Medical Center, Guangzhou Medical University, Guangzhou, 510623 Guangdong China

**Keywords:** Gestational diabetes mellitus, Gestational weight gain rate, Institute of Medicine, Fetal growth

## Abstract

**Background:**

Controversial evidence regarding the applicability of the IOM’s gestational weight gain (GWG) targets for women with gestational diabetes mellitus (GDM) has been reported. However, little is known about the weight gain rate (WGR) during the second and third trimesters. Moreover, previous studies failed to assess the effect modification of pre-pregnancy BMI because of the limited sample size. Therefore, we aimed to assess the applicability of the IOM recommendation for the WGR in women with GDM in different pre-pregnancy BMI categories.

**Methods:**

We conducted this retrospective cohort study of 5275 women with GDM who delivered at Guangzhou Women and Children’s Medical Center (GWCMC) between January 2017 and January 2021. Demographic and clinical information was collected from the electronic medical record system. The primary exposure was the WGR in the late second and third trimesters; they were classified as below, within, and above the IOM standard. The outcomes were fetal growth indicators, including large-for-gestational-age (LGA), macrosomia, small-for-gestational-age (SGA), and low birth weight (LBW). The associations between the WGR and such outcomes were assessed using multiple logistic regression.

**Results:**

A WGR below the IOM standard was associated with the decreased odds of LGA (adjusted OR 0.74; 95% CI 0.49–1.13) and macrosomia (adjusted OR 0.54, 95% CI 0.32–0.92) for women with GDM in the normal weight BMI class. Such decreases were observed greater for women with GDM in the overweight/obese class, with adjusted ORs of 0.34 (95% CI 0.09–0.88) for LGA and 0.31 (95% CI 0.01–0.84) for macrosomia, respectively. No significant difference was observed in the odds ratios of SGA and LBW across the different WGR groups.

**Conclusion:**

LGA and macrosomia are the main outcomes associated with the WGR in the late second and third trimesters, and a WGR below the IOM standard was associated with a decreased odds of such outcomes compared with a WGR within the IOM standard in women with GDM in the normal weight and overweight/obese classes. Our findings suggest that a stricter WGR target than that of the current IOM standard may be more beneficial for women with GDM.

**Supplementary Information:**

The online version contains supplementary material available at 10.1186/s12884-022-04762-4.

## Introduction

Gestational diabetes mellitus (GDM) has been a growing health concern and affects approximately 15% of pregnancies worldwide [1, 2]. GDM has been related to substantial short- and long-term adverse health outcomes such as the increased risk of large birth weight infants, obstructed labor, and the development of T2DM later in life [3–6]. Moreover, the offspring with large birth weight also have a high risk of developing obesity, impaired glucose tolerance, and type 2 diabetes in adulthood [7, 8]. In addition to GDM, gestational weight gain (GWG) is another well-known predictor for the short- and long-term health of a pregnant woman and her baby [9–12]. More importantly, women with GDM demonstrated a greater risk of adverse birth outcomes than women with normal glucose tolerance (NGT) from excessive weight gain [13], highlighting the urgent need for appropriate weight management for these women.

As no GDM-specific weight management guidelines currently exist, the 2009 weight recommendation established by the Institute of Medicine (IOM) (now the US National Academy of Medicine, NAM) has been suggested to be incorporated in the management of GDM pregnancy [14]. For example, women who are of normal weight are recommended to have a GWG of 11.5–16.0 kg and a weight gain rate (WGR) of 0.35–0.50 kg/wk in the second and third trimesters. Although such targets help guide weight management for women affected by GDM, attention has been drawn to its applicability. Women with GDM often follow a relatively restricted dietary and lifestyle management plan after diagnosis of this condition, and thus, their energy intake and weight gain differ greatly in from women with NGT [15–17]. In several recent retrospective cohort studies, the IOM recommendation for defining GWG targets has been shown to be less rigorous for women with GDM [18–21]. Unfortunately, GWG inherently relies on the length of the pregnancy, and a clinician is unable to predict the gestation length as well as if a woman will develop GDM at the beginning of pregnancy [22]. In addition, weight gain prior to the diagnosis of GDM has been fixed and cannot be changed. As such, monitoring the WGR in the late second and third trimesters, in contrast to GWG, would be more desirable for the management of weight for women with GDM. However, evidence on the applicability of the WGR targets for women with GDM was limited and conflicting [23–26]. Moreover, owing to the limited sample size, previous studies failed to assess the effect modification of pre-pregnancy BMI, although it is an important confounder for weight gain and adverse health outcomes [23–26].

Therefore, in this large retrospective cohort using real-world electronic health data, we aim to assess the applicability of the IOM’s WGR targets for women with GDM and further explore an alternative cut-off of the WGR for the benefit of fetal growth.

## Methods

This retrospective cohort study included pregnancies affected by GDM who gave birth between January 2017 and January 2021 at Guangzhou Women and Children’s Medical Center (GWCMC) which is one of the largest maternity services in South China, Guangzhou [27]. Per Chinese obstetric guidelines, all pregnant women follow a routine prenatal care protocol and schedule of frequent visits with the health system to identify risk factors and initiate preventive care measures [28]. For women indicating an intention to deliver at GWCMC, these prenatal visits are scheduled every two to four weeks up to 34 weeks and weekly thereafter, with lab tests and physical examinations performed and medical data captured by the integrated electronic medical record (EMR) system.

**Fig. 1:**
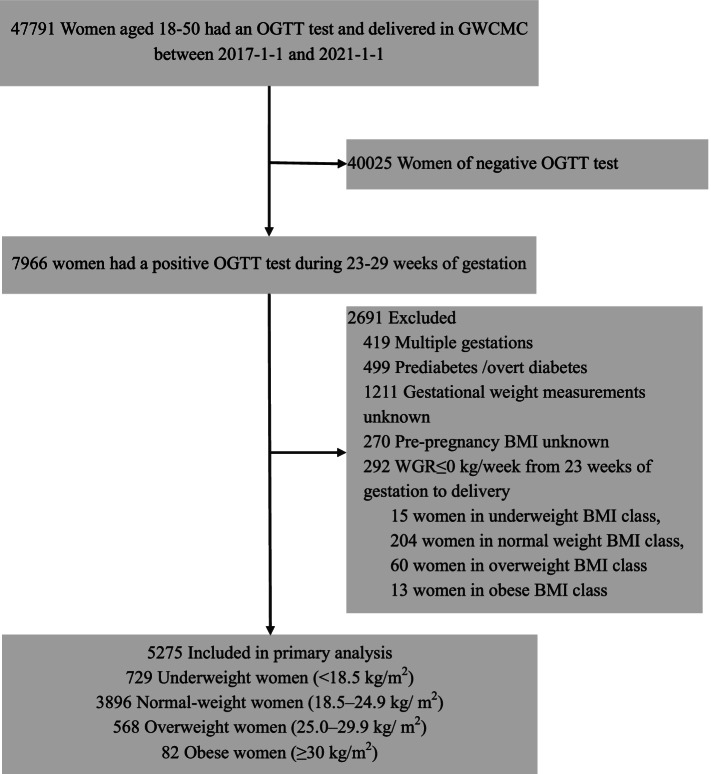
Derivation of the study population

Eligible participants were pregnant women aged 18–50 years who delivered at GWCMC and were diagnosed with GDM based on a 2-h 75-g oral glucose tolerance test (OGTT) during the gestational 23–28th weeks using the International Association of Diabetes and Pregnancy Study Groups criteria (fasting ≥ 5.1 mmol/L, or 1-h or ≥ 10.0 mmol/L, or 2-h ≥ 8.5 mmol/L) [29]. The exclusion criteria were as follows: 1) multiple gestations, 2) having a clinical diagnosis of pregestational diabetes mellitus (PGDM) or having overt diabetes (fasting plasma glucose (FBG) ≥ 7.0 mmol/l, or 2-h ≥ 11.0 mmol/L), and 3) unavailable data on maternal weight measurements or pre-pregnancy BMI. We also excluded women with WGR ≤ 0 in the second and third trimesters because pregnant women are generally not suggested to lose weight during pregnancy, especially in the second and third trimesters, given the potential risk of inadequate nutritional intake. The final study analysis included a total of 5275 women affected by GDM. There were 729 women with GDM in the underweight prepregnancy BMI class, 3896 women in the normal weight BMI class, 568 women in the overweight BMI class, and 82 in the obese BMI class (Fig. 1). Given all maternal and neonatal data were extracted from the hospital EMR system by a unique identifier with no participant involved in the design, the written informed consents were waived. This study was approved by the Ethics Committee of Guangzhou Women and Children’s Medical Center.

### Exposure assessment

The primary exposure was WGR in the late second and third trimesters, which was defined as the weight measurement at the last prenatal visit prior to delivery minus that at the prenatal visit within 23–28 weeks of gestation nearest to the OGTT test and then divided by the total number of weeks between these two visits. Maternal weight was measured in lightweight indoor clothing using calibrated electronic scales and captured automatically in the EMR system by a unique membership identifier used for prenatal care. We extracted these predefined weight measurements from the EMR system.

### Outcome assessment

The primary outcome of interest was fetal overgrowth including large-for-gestational-age infants (LGA, birthweight larger than the 90th percentile for gestational age by gender according to the 2015 Chinese sex- and gestational age-specific birthweight standards [30]) and macrosomia (birth weight ≥ 4000 g) [31]. Gestational age was confirmed by ultrasound examination in the first or second trimester. The second outcome was fetal undergrowth including small-for-gestational-age infants (SGA, birthweight smaller than the 10th percentile for gestational age by sex [30]) and low birth weight infants (LBW, birth weight less than 2500 g [32]). Infant birth weight was measured by midwives immediately after birth, and data was extracted from the EMR system.

### Covariate assessment

Baseline demographic and clinical characteristics were considered as potential confounders, including maternal age at delivery, parity (1, ≥ 2), levels of education (high school or below, college or university, and postgraduate), infant sex, family history of diabetes (yes/no) and prenatal hospital admission due to maternal complications (yes/no). In addition, weight-related variables were main confounders to be controlled; these included pre-pregnancy body mass index (BMI, kg/m2), weight gain prior to the late second trimester (prior weight gain, PWG), and the corresponding gestational length of PWG. PWG was calculated as the weight measurement at the prenatal visit within 23–28 weeks of gestation nearest to the OGTT test minus the self-reported preconception weight. Prepregnancy BMI was calculated from the self-reported preconception weight and the measured height during pregnancy as weight (in kg) divided by the height (in m) squared and classified as underweight (< 18.5 kg/m^2^), normal weight (18.5–24.9 kg/m2), overweight (25.0–29.9 kg/m2) and obese (≥ 30.0 kg/m2), respectively [33]. Since only 82 women were affected by obesity, these women and the women in the overweight class were combined into one category.

### Statistical analyses

Demographic and clinical variables were summarized for the whole cohort and compared according to their prepregnancy BMI using analysis of variance for continuous variables or the chi-square test for categorical variables. The WGR was classified into quintiles, with the lowest quintile regarded as the reference level, allowing for the assessment of the dose–response association between fetal growth and the WGR across underweight, normal weight and overweight/obese BMI classes, using the logistic regression model with the unadjusted and multivariable-adjusted odds ratios (ORs) and 95% confidence intervals (CIs) estimated. *P* for trend was obtained by treating the median of each group as a continuous variable. Covariates in the adjusted model included maternal age, education, parity, infant sex, family history of diabetes, hospital admission, pre-pregnancy BMI, PWG, and the corresponding gestational length of PWG.

To examine whether the IOM standard was applicable to women with GDM, the study population was divided into those below the IOM standard, those within the IOM standard, and those above the IOM standard according to the proposed thresholds (Table S1), with odds ratios of fetal growth estimated for women below the IOM standard and women above the IOM standard versus those within the IOM standard [13]. To test the robustness of the results, we examined the applicability of the IOM standard by excluding the patients with a family history of DM and hospital admission. A COVID outbreak through began near the end of 2019, and a lockdown began in 2020. Thus, we performed a sensitivity analysis by excluding women who delivered after the year of 2020 to avoid the bias from the changes in clinical practices. We also ran a sensitivity analysis for women in the overweight/obese BMI class by further including those women with WGR ≤ 0 in the second and third trimesters because weight loss was possibly beneficial for them. Moreover, we intentionally performed an explorative analysis for possible alternative ranges for the WGR based on the absolute risk of fetal overgrowth, by grouping the WGR into categories of a unit of 0.1 kg/wk ranging from 0 to 0.7 kg/wk or greater. The greater rates were not further categorized because of the limited number included. We compared the WGR range obtained from the explorative analysis with the IOM standard for the benefit of fetal growth. Accounting for the bias by unmeasured confounders, we ran a bias analysis referring to the previous study by Pasternak et al. [34], by constructing a hypothetical unmeasured confounder which decreases the risk of macrosomia by a factor of 0.1–0.9 and which has a 1–2 times higher proportion in women below IOM standard (with proportions varying from 10 to 90%) than women within IOM standard (varying from 5–45%). All analyses were performed using the statistical software program R, version 4.0.2. *P* < 0.05 was considered to indicate statistical significance.

## Results

### Characteristics of the study population

Table 1 summarizes the demographic and clinical variables of the whole cohort and those for the pre-pregnancy BMI categories. The final analysis consisted of 5275 pregnant women affected by GDM (mean [SD] maternal age, 32.8 [4.4] years), of whom 227 (4.3%) delivered an LGA infant, 154 (2.9%) delivered an infant with macrosomia, 680 (12.9%) delivered an SGA infant, and 198 (3.8%) delivered an LBW infant. Women with GDM, on average, had a WGR of 0.37 (SD 0.19) kg/wk in the late second and third trimesters. Of these women, 2576 (48.8) were below the IOM standard, 1398 (26.5) within the IOM standard, and 1301 (24.7) above the IOM standard. Women with GDM in different BMI categories varied greatly in baseline and clinical characteristics. For example, compared with women with GDM in the normal weight BMI class, those women in the overweight/obese BMI class were more likely to be primiparous, have a lower education level, and have a higher PGW but lower WGR in the late second and third trimesters, however, the proportion of women with a WGR above the IOM recommendation (48.6%) was higher (22.5%) in the overweight/obese BMI class than that in the normal weight BMI class.

**Table 1:** Demographic and clinical characteristics of the study population stratified by prepregnancy BMI

Maternal and neonatal variables, mean (SD) or n (%)	Overall (*N* = 5275)	Underweight (*n* = 729)	Normal weight (*n* = 3896)	Overweight/Obese ^a^ (*n* = 650)	*P-*value
Maternal age, years	32.8 (4.4)	30.9 (4.0)	33.1 (4.4)	33.8 (4.4)	< 0.001
Education level					< 0.001
High school or below	722 (13.7)	87 (11.9)	523 (13.4)	112 (17.2)	
College or university	3547 (67.2)	520 (71.3)	2634 (67.6)	393 (60.5)	
Postgraduate	557 (10.6)	64 (8.8)	430 (11.0)	63 (9.7)	
Unknown	449 (8.5)	58 (8.0)	309 (7.9)	82 (12.6)	
Pre-pregnancy BMI, kg/m^2^					-
Underweight	729 (13.8)	729 (100)	-	-	
Normal weight	3896 (73.9)	-	3896 (100)	-	
Overweight	568 (10.8)	-	-	568 (87.4)	
Obese	82 (1.6)	-	-	82 (12.6)	
Family history of diabetes, yes	727 (13.8)	80 (11.0)	544 (14.0)	103 (15.8)	0.03
Parity, ≥ 2	2724 (51.6)	264 (36.2)	2069 (53.1)	391 (60.2)	< 0.001
PWG, kg	7.1(3.6)	7.5 (3.0)	7.2 (3.5)	5.6 (4.4)	< 0.001
Weeks of PWG, wk	25.5 (1.4)	25.6 (1.5)	25.5 (1.4)	25.4 (1.5)	0.05
Last prenatal weight, kg	66.7 (8.9)	57.5 (5.4)	66.4 (7.0)	79.0 (8.6)	< 0.001
Weeks of last weight, kg	38.5 (1.4)	38.52(1.4)	38.5 (1.4)	38.5 (1.3)	0.99
WGR in late 2^nd^ and 3^rd^ trimesters, wg/wk	0.37 (0.2)	0.39 (0.2)	0.37 (0.2)	0.35 (0.2)	< 0.001
Below IOM	2576 (48.8)	462 (63.4)	1918 (49.2)	196 (30.2)	< 0.001
Within IOM	1398 (26.5)	157 (21.5)	1103 (28.3)	138 (21.2)	
Above IOM	1301 (24.7)	110 (15.1)	875 (22.5)	316 (48.6)	
Hospital admission, yes	266 (5.0)	27 (3.7)	200 (5.1)	39 (6.0)	0.13
Sex, female	2472 (46.9)	356 (48.8)	1829 (46.9)	287 (44.2)	0.23
Birth weight, gram	3201 (413)	3061 (360)	3207 (409)	3324 (447)	< 0.001
Clinical outcomes					
LGA, yes	227 (4.3)	7 (1.0)	155 (4.0)	65 (10.0)	< 0.001
Macrosomia, yes	154 (2.9)	4 (0.5)	109 (2.8)	41 (6.3)	< 0.001
SGA, yes	680 (12.9)	164 (22.5)	469 (12.0)	47 (7.2)	< 0.001
LBW yes	198 (3.8)	31 (4.3)	150 (3.9)	17 (2.6)	0.231

*Abbreviations*: *PWG* Prior weight gain defined as weight gain prior to late 2^nd^ trimester, *WGR* Weight gain rate, *IOM* Institute of Medicine, *LGA* large-for-gestational-age infant, *SGA* Small-for-gestational-age infant, *LBW* Low birth weight

^a^Women with overweight and women with obesity were combined into one category because of the small number of subjects in the obese BMI class

### Association between the WGR in the late second and third trimesters and fetal growth

**Fig. 2:**
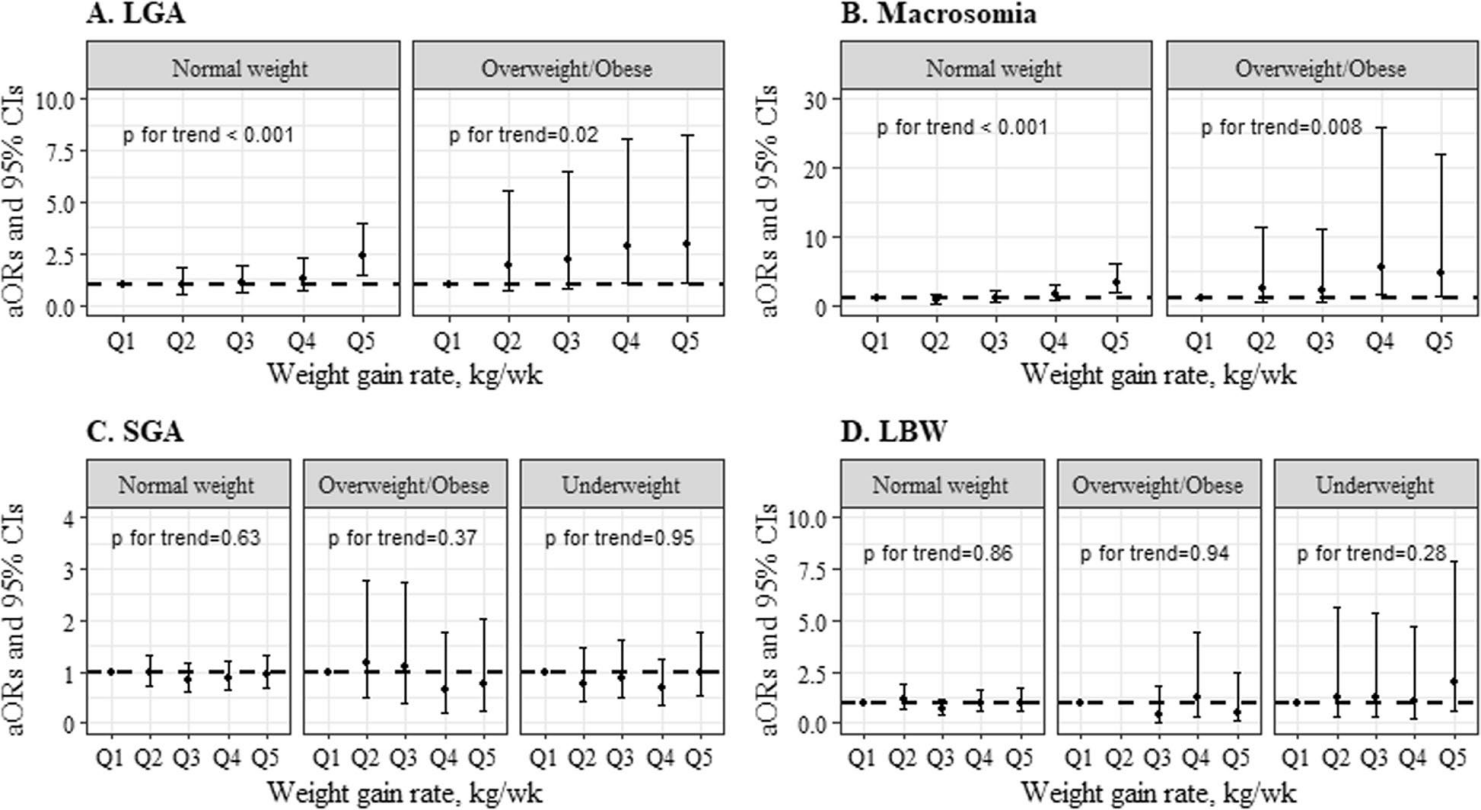
The association between fetal growth and the weight gain rate in quintiles of women with gestational diabetes mellitus by pre-pregnancy BMI. Abbreviations: aOR, adjusted odds risk; CI, confidence interval; LGA, large-for-gestational-age infant; SGA, small-for-gestational-age infant; LBW, low birth weight. Adjusted maternal age, education, parity, infant sex, family history of diabetes, hospital admission, PWG (weight gain prior to late 2^nd^ trimester), and the corresponding gestational length of PWG

Figure 2 depicts the dose–response association between fetal growth and the WGR for the quintiles of women with GDM by prepregnancy BMI. The odds ratios of LGA and macrosomia both increased progressively from the lowest to median quintiles to the highest quintile of the WGR (*P* for trend < 0.05). No significant association was observed between the WGR and fetal undergrowth including SGA and LBW.

Table 2 displays the odd ratios for fetal growth for women below and above the IOM standard compared with those women within the IOM standard within the same prepregnancy BMI class. No significant difference in the odds of SGA and LBW existed between women above or below the IOM standard and women within the IOM standard across each BMI category (Table 2). Instead, compared to women within the IOM standard, we observed a significantly increased odds of fetal overgrowth for women above the IOM standard (LGA: 1.88, 95% CI 1.25–2.87, macrosomia: 2.29, 95% CI 1.43–3.72) and a reduced odds of that for women below the IOM standard in the normal weight BMI class (LGA: 0.74, 95% CI 0.49–1.13; macrosomia: 0.54, 95% CI 0.32–0.92), but not significant for LGA. These decreases were greater for women below the IOM standard (LGA: 0.34, 95% CI 0.14–0.77; macrosomia: 0.31, 95% CI 0.09–0.88) in the overweight/obese BMI class. However, women above the IOM standard in the overweight/obese BMI class showed no difference in the odds of fetal overgrowth compared with those women within the IOM standard (Table 2).

**Table 2:** Association between the IOM-recommended weight gain rate and fetal growth among women with gestational diabetes mellitus stratified by prepregnancy BMI

Outcomes ^a, b^	Underweight (*n* = 729)		Normal weight (*n* = 3896)		Overweight/Obese (*n* = 650)	
	**Below (*n* = 462)**	**Above (*n* = 110)**	**Below (*n* = 1918)**	**Above (*n* = 875)**	**Below (*n* = 196)**	**Above (*n* = 316)**
**LGA**						
Cases (%)	3 (0.6) vs 0	4 (3.6) vs 0	54 (2.8) vs 41 (3.7)	60 (6.9) vs 41(3.7)	2 (1.7) vs 27 (12.2)	36 (11.7) vs 27 (12.2)
OR (95% CI)	-	-	0.75 (0.50–1.14)	1.91 (1.27–2.88)	0.32 (0.13–0.72)	0.91 (0.51–1.69)
aOR (95% CI)	-	-	0.74 (0.49–1.13)	1.88 (1.25–2.87)	0.34 (0.14–0.77)	0.90 (0.49–1.70)
**Macrosomia**						
Cases (%)	1 (0.2) vs 0	3 (2.7) vs 0	29 (1.5) vs 29 (2.6)	51(5.8) vs 29 (2.6)	5 (2.6) vs 11 (8.0)	25 (7.9) vs 11 (8.0)
OR (95% CI)	-	-	0.57 (0.34–0.96)	2.29 (1.45–3.69)	0.30 (0.09–0.85)	0.99 (0.48–2.16)
aOR (95% CI)	-	-	0.54 (0.32–0.92)	2.29 (1.43–3.72)	0.31 (0.09–0.88)	1.03 (0.49–2.28)
**SGA**						
Cases (%)	108 (23.4) vs 29 (18.5)	27 (24.5) vs 29 (18.5)	241(12.6) vs 121(11.0)	107(12.2) vs 121(11.0)	17 (8.7) vs 11 (8.0)	19 (6) vs 11 (8.0)
OR (95% CI)	1.35 (0.86–2.16)	1.44 (0.79–2.60)	1.17 (0.93–1.47)	1.13 (0.86–1.49)	1.10 (0.50–2.49)	0.74 (0.35–1.65)
aOR (95% CI)	1.44 (0.91–2.34)	1.60 (0.87–2.97)	1.16 (0.92–1.47)	1.10 (0.83–1.46)	1.03 (0.47–2.35)	0.71 (0.33–1.58)
**LBW**						
Cases (%)	18 (3.9) vs 6 (3.8)	7 (6.4) vs 6 (3.8)	78 (4.1) vs 37 (3.4)	35 (4.0) vs 37 (3.4)	6 (3.1) vs 1 (0.7)	10 (3.2) vs 1 (0.7)
OR (95% CI)	1.02 (0.42–2.86)	1.71 (0.55–5.45)	1.22 (0.83–1.84)	1.21 (0.81–1.82)	4.33 (0.73–82.2)	4.48 (0.85–82.6)
aOR (95% CI)	1.13 (0.44–3.32)	1.64 (0.50–5.52)	1.20 (0.75–1.92)	1.21 (0.75–1.94)	4.77 (0.78–91.8)	4.65 (0.85–86.7)

*Abbreviations*: *IOM* Institute of Medicine, *OR* Odds ratio, *aOR* Adjusted odds ratio, *CI* Confidence interval, *LGA* Large-for-gestational-age infant, *SGA* Small-for-gestational-age infant, *LBW* Low birth weight

^a^The reference group was women who had a WGR within IOM recommendation

^b^Adjusted maternal age, education, parity, infant sex, family history of diabetes, hospital admission, PWG (weight gain prior to late 2^nd^ trimester), and the corresponding gestational length of PWG

### Sensitivity and explorative analyses

The main findings of association between WGR and fetal overgrowth and undergrowth were generally consistent in sensitivity analyses restricting the populations to those without a family history of diabetes and hospital admission, excluding the year 2020 to account for the influence of COVID-19 (Table S2), and including women with WGR ≤ 0 in the overweight/obese BMI class (Table S3). The adjusted estimates of macrosomia considering unmeasured confounders are displayed in Table S4. In the main analysis, the adjusted odds of macrosomia in women below the IOM standard was 0.54 compared with those women within the IOM standard. Only when the odds of macrosomia associated with the confounder was as lower as 0.1-fold and the prevalence of the confounder reaches 35% in women within the IOM standard and 70% in women below the IOM standard could the association change to null (Table S4). In our view, there would be a low possibility of such a hypothetical confounder. Consequently, our finding was less likely to be influenced by the unmeasured confounder.

In the explorative analysis for possible WGR range alternatives, the WGRs of 0–0.3, and 0–0.2 were possible ranges for women with GDM in the normal weight and overweight classes, respectively. Exceeding these ranges tended to stably or sharply increase the risks of fetal overgrowth (Figure S1). The comparison between such explorative ranges and the IOM targets is presented in Table S5. Women with GDM who had a WGR below 0.3 kg/wk in the normal weight class showed a decreased odds of fetal overgrowth versus those within the IOM standard (LGA: aOR 0.76, 95% CI 0.49–1.18, macrosomia: aOR 0.56, 95% CI 0.32–0.98). Such decrease in the odds of fetal growth was more obvious in women with GDM who had a WGR below 0.2 kg/wk in the overweight class (LGA: aOR 0.34, 0.10–0.98) and macrosomia: aOR 0.11, 0.01–0.73) (Table S5). Women with GDM in the underweight and obese classes were not analyzed due to the small number of subjects in these categories.

## Discussion

In this prospectively collected clinical data from 5275 women affected by GDM, fetal overgrowth, including LGA and macrosomia, was the main adverse birth outcome associated with the WGR in the late second and third trimesters. There was no significant association between fetal undergrowth and the WGR. Women below the IOM standard showed a 26% and 46% reduction in the odds of LGA, 66% and 69% reduction in that of macrosomia in the normal weight and overweight/obese BMI class, respectively, versus those women within the IOM standard. Women in the underweight BMI class were unable to be analyzed owing to limited cases in such pregnancies. Our findings underline the importance of a stricter WGR target to manage weight for women with GDM given the fetal growth risk, especially for those women affected by both GDM and overweight/obesity.

The association between the WGR after the diagnosis of GDM and fetal overgrowth has been previously reported. In a study of 635 women affected by GDM, Harper and his colleagues noticed a 1.36-fold higher odds of macrosomia and a 1.40-fold odds of LGA for every 1-lb/wk increase in weight gain after the diagnosis of GDM [22]. This finding is consistent with the findings of Zheng and his colleagues [17]. Such findings suggest the importance of appropriate weight management for these women. For decades, the IOM recommendations from the general pregnant population have been used to guide weight management in GDM pregnancies [13]. However, using these recommendations has the potential to skew the distribution of GDM pregnancies to insufficient weight gain. In our study, only 28.1% of women were within normal range and 47.4% did not gain sufficient weight, which is more than twice that of the general pregnant population (20.9%) [35]. This phenomenon was consistently observed in many studies, with a reported percentage of insufficient weight gain of 31–50.3% using GWG targets [19–21] and that of 28–40.1% using WGR targets [18, 23, 24]. More recently, a study of 1138 women with NGT and 1200 women affected by GDM, reported a similar increase in the odds of fetal overgrowth in women with GDM who were within GWG targets (1.42, 95% CI 1.03–1.95) and women with NGT who were above the GWG targets (1.47, 95% CI 1.02–2.13), as compared with NGT women within the GWG targets [20], suggesting that the weight gain targets should be more stringent for women with GDM than for women with NGT.

Indeed, the evidence regarding the applicability of the IOM GWG targets to women with GDM [18–21] is controversial. For example, Xu et al. noted a significant decrease in fetal overgrowth risk by following a more stringent GWG target (minus 1 or 2 kg) [20]. In a historical cohort study of 481 obese women, Jensen et al. suggested that a strict weight gain of 5–10 kg lower than the IOM recommendations was more favorable for fetal growth [33]. However, since weight gain cannot be altered prior to the diagnosis of GDM, the second and third trimesters are the critical periods for women with GDM. Thus, the WGR during this period rather than total GWG targets would be more appropriate to manage weight in these women. Data on the WGR in the second and third trimesters are currently limited. In a study of 1606 women with GDM, Shi et al. found that women with a WGR below the IOM standard had lower odds ratios for LGA and macrosomia than those women within the IOM standard [24]. In contrast, in a study of 593 Indian women with GDM, Kashyap et al. found no statistical difference in the prevalence of LGA and SGA across groups below, within, and above the WGR targets [26], which is similar to the findings of Kurtzhals et al. [25] and Harper et al. [23] in white women. However, a certain limitation in these studies is that they failed to test the hypothesis across different BMI categories due to the limited sample size, even though BMI has been reported to be a key important confounder for gestational weight gain [36, 37]. Moreover, they were also unable to determine the applicability of the IOM standard for each BMI category. In our study, WGR below the IOM standard showed no significant difference in the odds of SGA and LBW compared to WGR within the IOM standard across each BMI category. However, women with a WGR below the IOM standard showed lower odds of fetal overgrowth than WGR within the IOM standard in the normal weight BMI class. This finding was more pronounced in women in the overweight/obese BMI class. Moreover, in the explorative analysis to identify a possible alternative cutoff, we observed a significant reduction in the odds of fetal overgrowth by restricting the upper threshold of the WGR to 0.3 and 0.2 for women in the normal weight class and those in the overweight BMI class, respectively. Our new evidence supports a need for rigorous WGR targets to optimize the weight management of GDM pregnancies. Women affected by GDM are typically educated more on nutrition knowledge and follow a more restricted diet management plan to control the glycemic status, which consequently results in a greater reduction in energy intake, carbohydrate intake as well as weight gain compared to women with NGT [13]. This may be a possible explanation for why stringent weight gain targets was prefered for women with GDM.

The main strengths of this study included the large sample size and the prospective nature of the clinical data collection. Additionally, the extracted maternal weight measurements based on the EMR system were weighed using the same weighing scale, which has a higher internal consistency compared with self-reported data. Thus, exposure misclassification was largely avoided. There were also some limitations in this study. First, despite the large scale on the whole cohort, the number of patients in the underweight and overweight/obese BMI categories was small. Therefore, large data for these categories are warranted to replicate our findings. Second, there were inevitably some unmeasured confounders in this study as they were not captured in the EMR system including household income, and smoking status. However, the results remained stable after adjustment for unmeasured confounders in the bias analysis, thereby our findings were unlikely to be influenced. Third, this study was not pointed to determine the optimal target for WGR, although a specified range lower than the IOM standard was found with a strong statistical power to decrease fetal overgrowth risk. To define an optimal weight target, more relevant mother and child outcomes should often be considered as much as possible. The outcome in the present study was fetal growth only, and future research considering other potential clinical outcomes are needed to advance our findings toward an optimal WGR target before they can be applied in clinical practice. Last, the population in this study consisted of Han Chinese individuals, whose children have higher sex- and gestational age-specific birthweight cutoffs to define LGA compared to the white population, as a result, the prevalence of LGA newborn in our study population was lower than that in the white population [30, 38]. Given this, our findings should be extrapolated beyond this context with caution. Moreover, the Asian population tends to have lower BMI cutoffs than the white population when identifying high-risk individuals and taking corresponding interventions. However, since we focused on the applicability of the IOM standard, it may be more appropriate for this study to use a BMI threshold consistent with the IOM recommendation than the Asian-specific one. Nonetheless, this is a limitation in our study, and applying Asian-specific BMI cutoffs is warranted in future studies to determine optimal target for such populations.

## Conclusion

A WGR below the IOM standard was associated with a decreased odds of fetal overgrowth compared with a WGR within the IOM standard in women with GDM in the normal weight and overweight/obese classes. These findings suggest the need for a stricter WGR target in the late second and third trimesters for these women.

## Supplementary Information

**Additional file 1.**

## Data Availability

The datasets generated and analyzed during the current study are not publicly available due to ethical concerns but are available from the corresponding author on reasonable request.
