# Supplementary material for: Weight gain rate in the second and third trimesters and fetal growth in women with gestational diabetes mellitus: a retrospective cohort study
Source: BMC Pregnancy Childbirth. 2022 May 20;22:424. doi: 10.1186/s12884-022-04762-4 (PMC9122248; doi:10.1186/s12884-022-04762-4)

**Supplementary tables and figures**

Table S1. 2009 Institute of Medicine (IOM) recommendations for weight gain rate (WGR) during pregnancy, by prepregnancy BMI

Table S2. Sensitivity analysis of the association between the WGR in late 2^nd^ and 3^rd^ trimesters and fetal growth for women with GDM, stratified by prepregnancy BMI

Table S3. Sensitivity analysis of the association between the WGR in late 2^nd^ and 3^rd^ trimesters and fetal growth for women with GDM in overweight/obese BMI class, further including women with WGR≤0

Table S4. Array approach sensitivity analysis of the applicability of IOM standard to normal-weight women with GDM based on macrosomia risk, adjusting for unmeasured confounder

Table S5. Comparison between the IOM’s WGR targets and explorative ranges stratified by prepregnancy BMI

Figure S1 The absolute risk of fetal growth across the weight gain rate in late second and third trimesters

Table S1. 2009 Institute of Medicine (IOM) recommendations for weight gain rate (WGR) during pregnancy, by pre-pregnancy BMI

| Prepregnancy BMI | Weight gain rate in Second and Third Trimesters, Mean (Range), kg/wk |
| --- | --- |
| Underweight (<18.5 kg/m2) | 0.51 (0.44–0.58) |
| Normal weight (18.5–24.9 kg/m2) | 0.42 (0.35–0.50) |
| Overweight (25.0–29.9 kg/m2) | 0.28 (0.23–0.33) |
| Obese (>=30.0 kg/m2) | 0.22 (0.17–0.27) |

Table S2. Sensitivity analysis of the association between the WGR in late 2^nd^ and 3^rd^ trimesters and fetal growth for women with GDM, stratified by prepregnancy BMI

| **Subgroups** | **Underweight** | |  | **Normal weight** | |  | **Overweight/Obese** | |
| --- | --- | --- | --- | --- | --- | --- | --- | --- |
|  | **Case/N, %** | **Adjusted OR** |  | **Case/N, %** | **Adjusted OR** |  | **Case/N, %** | **Adjusted OR** |
| **Women who had no family history of GDM and no hospitalization** | | | | | | | | |
| **LGA** |  |  |  |  |  |  |  |  |
| Within IOM | 0/137 (0) | Ref |  | 37/898 (4.1) | Ref |  | 17/114 (14.9) | Ref |
| Below IOM | 3/394 (0.8) | - |  | 46/1554 (3.0) | 0.69 (0.45-1.09) |  | 6/157 (3.8) | 0.23 (0.08, 0.58) |
| Above IOM | 4/93 (4.3) | - |  | 51/725 (7.0) | 1.66 (1.07-2.59) |  | 29/245 (11.8) | 0.74 (0.39, 1.45) |
| **Macrosomia** |  |  |  |  |  |  |  |  |
| Within IOM | 0/137 (0) | Ref |  | 26/898 (2.9) | Ref |  | 11/114 (9.6) |  |
| Below IOM | 1/394 (0.3) | - |  | 25/1554 (1.6) | 0.53 (0.30-0.93) |  | 3/157 (1.9) | 0.19 (0.04, 0.62) |
| Above IOM | 3/93 (3.2) | - |  | 43/725 (5.9) | 1.98 (1.21-3.31) |  | 19/245 (7.8) | 0.79 (0.37, 1.80) |
| **SGA** |  |  |  |  |  |  |  |  |
| Within IOM | 29/137 (21.2) | Ref |  | 99/898 (11) | Ref |  | 7/114 (6.1) | Ref |
| Below IOM | 91/394 (23.1) | 1.14 (0.71-1.85) |  | 189/1554 (12.2) | 1.11 (0.86-1.44) |  | 13/157 (8.3) | 1.37 (0.54, 3.76) |
| Above IOM | 23/93 (24.7) | 1.37 (0.72-2.58) |  | 88/725 (12.1) | 1.14 (0.84-1.55) |  | 16/245 (6.5) | 1.07 (0.44, 2.86) |
| **LBW** |  |  |  |  |  |  |  |  |
| Within IOM | 6/137 (4.4) | Ref |  | 25/898 (2.8) | Ref |  | 0/114 (0) | Ref |
| Below IOM | 12/394 (3) | 0.67 (0.25-1.96) |  | 62/1554 (4) | 1.45 (0.91-2.36) |  | 6/157 (3.8) | - |
| Above IOM | 6/93 (6.5) | 1.61 (0.48-5.33) |  | 30/725 (4.1) | 1.54 (0.90-2.67) |  | 8/245 (3.3) | - |
| **Excluding the year of delivery of 2020** | | | | | | | | |
| **LGA** |  |  |  |  |  |  |  |  |
| Within IOM | 0/129 (0) | Ref |  | 33/888 (3.7) | Ref |  | 16/120 (13.3) | Ref |
| **Table S2 continued.** | | | | | | | | |
| Below IOM | 2/377 (0.5) | - |  | 43/1538 (2.8) | 0.74 (0.47-1.19) |  | 6/170 (3.5) | 0.24 (0.08, 0.62) |
| Above IOM | 3/80 (3.8) | - |  | 45/672 (6.7) | 1.73 (1.09-2.76) |  | 31/252 (12.3) | 0.90 (0.47, 1.77) |
| **Macrosomia** |  |  |  |  |  |  |  |  |
| Within IOM | 0/129 (0) | Ref |  | 22/888 (2.5) | Ref |  | 10/120 (8.3) | Ref |
| Below IOM | 0/377 (0) | - |  | 24/1538 (1.6) | 0.61 (0.34-1.11) |  | 3/170 (1.8) | 0.20 (0.04, 0.67) |
| Above IOM | 2/80 (2.5) | - |  | 40/672 (6) | 2.28 (1.35-3.95) |  | 20/252 (7.9) | 0.97 (0.44, 2.25) |
| **SGA** |  |  |  |  |  |  |  |  |
| Within IOM | 24/129 (18.6) | Ref |  | 94/888 (10.6) | Ref |  | 11/120 (9.2) | Ref |
| Below IOM | 91/377 (24.1) | 1.43 (0.87-2.41) |  | 197/1538 (12.8) | 1.22 (0.94-1.59) |  | 14/170 (8.2) | 0.88 (0.38, 2.05) |
| Above IOM | 18/80 (22.5) | 1.41 (0.69-2.81) |  | 79/672 (11.8) | 1.15 (0.83-1.58) |  | 15/252 (6) | 0.61 (0.27, 1.42) |
| **LBW** |  |  |  |  |  |  |  |  |
| Within IOM | 5/129 (3.9) | Ref |  | 30/888 (3.4) | Ref |  | 1/120 (0.8) | Ref |
| Below IOM | 15/377 (4) | 1.04 (0.39-3.26) |  | 66/1538 (4.3) | 1.27 (0.83-2.00) |  | 6/170 (3.5) | 4.31 (0.72, 82.1) |
| Above IOM | 4/80 (5) | 1.42 (0.34-5.60) |  | 22/672 (3.3) | 1.00 (0.57-1.75) |  | 10/252 (4) | 4.87 (0.92, 90.0) |

Abbreviations: WGR, weight gain rate; IOM, Institute of Medicine; LGA, large-for-gestational-age infant; SGA, small-for-gestational-age infant; LBW, low birth weight. ^a^ The reference group was women had a WGR within IOM recommendation; ^b^ Adjusted PWG, and the corresponding gestational length of PWG.

Table S3. Sensitivity analysis of the association between the WGR in late 2nd and 3rd trimesters and fetal growth for women with GDM in overweight/obese BMI class, further including women with WGR≤0

| **Outcomes ^a, b^** |  | **Overweight/Obese (n=723)** | | |
| --- | --- | --- | --- | --- |
|  |  | **Below (n=**269**)** | **Within (n=138)** | **Above (n=316)** |
| **LGA** |  |  |  |  |
| Cases (%) |  | 12 (4.5) | 18 (13.0) | 38 (12.0) |
| OR (95% CI) |  | 0.31 (0.14, 0.66) | 1 | 0.91 (0.51, 1.69) |
| aOR (95% CI) |  | 0.32 (0.13, 0.76) | 1 | 0.95 (0.49, 1.95) |
| **Macrosomia** |  |  |  |  |
| Cases (%) |  | 7 (2.6) | 11(8.0) | 25 (7.9) |
| OR (95% CI) |  | 0.31 (0.11, 0.80) | 1 | 0.99 (0.48, 2.16) |
| aOR (95% CI) |  | 0.38 (0.13, 1.13) | 1 | 1.07 (0.46, 2.71) |
| **SGA** |  |  |  |  |
| Cases (%) |  | 23 (8.6) | 11 (8.0) | 19 (6.0) |
| OR (95% CI) |  | 1.08 (0.52, 2.37) | 1 | 0.74 (0.35, 1.65) |
| aOR (95% CI) |  | 1.09 (0.51, 2.51) | 1 | 0.60 (0.26, 1.42) |
| **LBW** |  |  |  |  |
| Cases (%) |  | 9 (3.3) | 1 (0.7) | 10 (3.2) |
| OR (95% CI) |  | 4.74 (0.88, 87.91) | 1 | 4.48 (0.85, 82.57) |
| aOR (95% CI) |  | 4.90 (0.88, 91.90) | 1 | 3.46 (0.61, 65.18) |

^a^ The reference group was women who had a WGR within IOM recommendation.

^b^ Adjusted maternal age, education, parity, infant sex, family history of diabetes, hospital admission, PWG (weight gain prior to late 2nd trimester), and the corresponding gestational length of PWG.

Table S4. Array approach sensitivity analysis of the applicability of IOM standard to normal-weight women with GDM based on macrosomia risk, adjusting for unmeasured confounder

| Adjusted RR_M_ | Adjusted RR_UC_ | RR_CD_ | Prevalence of unmeasured confounder | | Bias |
| --- | --- | --- | --- | --- | --- |
|  |  |  | Within IOM (PC0) | Below IOM (PC1) |  |
| 0.54 | 0.54 | 0.9 | 5.0% | 10.0% | 0.0% |
| 0.54 | 0.55 | 0.8 | 5.0% | 10.0% | 0.0% |
| 0.54 | 0.55 | 0.7 | 5.0% | 10.0% | 0.0% |
| 0.54 | 0.55 | 0.6 | 5.0% | 10.0% | 0.0% |
| 0.54 | 0.55 | 0.5 | 5.0% | 10.0% | 0.0% |
| 0.54 | 0.56 | 0.4 | 5.0% | 10.0% | 0.0% |
| 0.54 | 0.56 | 0.3 | 5.0% | 10.0% | 0.0% |
| 0.54 | 0.56 | 0.2 | 5.0% | 10.0% | 0.0% |
| 0.54 | 0.57 | 0.1 | 5.0% | 10.0% | 0.0% |
| 0.54 | 0.55 | 0.9 | 10.0% | 20.0% | 0.0% |
| 0.54 | 0.55 | 0.8 | 10.0% | 20.0% | 0.0% |
| 0.54 | 0.56 | 0.7 | 10.0% | 20.0% | 0.0% |
| 0.54 | 0.56 | 0.6 | 10.0% | 20.0% | 0.0% |
| 0.54 | 0.57 | 0.5 | 10.0% | 20.0% | 10.0% |
| 0.54 | 0.58 | 0.4 | 10.0% | 20.0% | 10.0% |
| 0.54 | 0.58 | 0.3 | 10.0% | 20.0% | 10.0% |
| 0.54 | 0.59 | 0.2 | 10.0% | 20.0% | 10.0% |
| 0.54 | 0.60 | 0.1 | 10.0% | 20.0% | 10.0% |
| 0.54 | 0.55 | 0.9 | 20.0% | 40.0% | 0.0% |
| 0.54 | 0.56 | 0.8 | 20.0% | 40.0% | 0.0% |
| 0.54 | 0.58 | 0.7 | 20.0% | 40.0% | 10.0% |
| 0.54 | 0.59 | 0.6 | 20.0% | 40.0% | 10.0% |
| 0.54 | 0.61 | 0.5 | 20.0% | 40.0% | 10.0% |
| 0.54 | 0.63 | 0.4 | 20.0% | 40.0% | 10.0% |
| 0.54 | 0.65 | 0.3 | 20.0% | 40.0% | 20.0% |
| 0.54 | 0.67 | 0.2 | 20.0% | 40.0% | 20.0% |
| 0.54 | 0.69 | 0.1 | 20.0% | 40.0% | 20.0% |
| 0.54 | 0.56 | 0.9 | 30.0% | 60.0% | 0.0% |
| 0.54 | 0.58 | 0.8 | 30.0% | 60.0% | 10.0% |
| 0.54 | 0.60 | 0.7 | 30.0% | 60.0% | 10.0% |
| 0.54 | 0.63 | 0.6 | 30.0% | 60.0% | 10.0% |
| 0.54 | 0.66 | 0.5 | 30.0% | 60.0% | 20.0% |
| 0.54 | 0.69 | 0.4 | 30.0% | 60.0% | 20.0% |
| 0.54 | 0.74 | 0.3 | 30.0% | 60.0% | 30.0% |
| 0.54 | 0.79 | 0.2 | 30.0% | 60.0% | 30.0% |
| 0.54 | 0.86 | 0.1 | 30.0% | 60.0% | 40.0% |
| 0.54 | 1.00 | 0.1 | 35.0% | 70.0% | 50.0% |
| 0.54 | 1.23 | 0.1 | 40.0% | 80.0% | 60.0% |
| 0.54 | 1.69 | 0.1 | 45.0% | 90.0% | 70.0% |

Notes: Scenario: Confounder decrease risk of macrosomia with 2 times higher proportion among normal weight women below IOM standard than those within IOM standard.

RR_CD_: association between confounder and outcome; PC0: prevalence of confounder in group of women within IOM; PC1: prevalence of confounder in group of women below IOM; Adjusted RR_M_: adjusted odds risk estimated in the main analysis; adjusted RR_UC_: odds risk when adjusted for unmeasured confounder further, Adjusted RR_UC_ =Adjusted RR_M_ / { (PC1*(RR_CD_-1)+1) / (PC0*(RRCD-1)+1) }; Bias: Per cent bias introduced by not adjusting for confounder, Bias = ((Adjusted RR_UC_ - Adjusted RR_M_)/ Adjusted RR_UC_)*100

Table S5. Comparison between the IOM’s WGR targets and explorative ranges stratified by prepregnancy BMI

| **Outcomes** | **Normal weight (n=2594)** | | |  | **Overweight (n=255)** | | |
| --- | --- | --- | --- | --- | --- | --- | --- |
|  | **Case/N (%)** | **Unadjusted OR** | **Adjusted OR** ^a^ |  | **Case/N (%)** | **Unadjusted OR** | **Adjusted OR** ^a^ |
| **LGA** |  |  |  |  |  |  |  |
| Within IOM | 41/1103 (4.0) | Ref | Ref |  | 14/121 (11.6) | Ref | Ref |
| Within explorative range | 45/1491 (3.0) | 0.81 (0.52-1.24) | 0.78 (0.50-1.20) |  | 5/134 (3.7) | 0.30 (0.09-0.80) | 0.30 (0.09-0.81) |
| **Macrosomia** |  |  |  |  |  |  |  |
| Within IOM | 29/1103 (2.6) | Ref | Ref |  | 7/121 (5.8) | Ref | Ref |
| Within explorative range | 24/1491 (1.6) | 0.61 (0.35-1.05) | 0.57 (0.33-0.99) |  | 1/134 (0.7) | 0.12 (0.01-0.70) | 0.12 (0.01-0.70) |
| **SGA** |  |  |  |  |  |  |  |
| Within IOM | 121/1103 (11.0) | Ref | Ref |  | 10/121 (8.3) | Ref | Ref |
| Within explorative range | 187/1491 (12.5) | 1.16 (0.91-1.49) | 1.16 (0.91-1.48) |  | 11/134 (8.2) | 0.99 (0.40-2.47) | 0.97 (0.39-2.44) |
| **LBW** |  |  |  |  |  |  |  |
| Within IOM | 37/1103 (3.4) | Ref | Ref |  | 1/121 (0.8) | Ref | Ref |
| Within explorative range | 66/1491 (4.4) | 1.33 (0.89-2.03) | 1.35 (0.90-2.05) |  | 6/134 (4.5) | 5.62 (0.94-107.1) | 5.42 (0.90-103.5) |

Abbreviations: IOM, Institute of Medicine; LGA, large-for-gestational-age infant; SGA, small-for-gestational-age infant; LBW, low birth weight. ^a^ Adjusted PWG, and the corresponding gestational length of PWG.

Figure S1 The absolute risk of fetal growth across the weight gain rate in late second and third trimesters


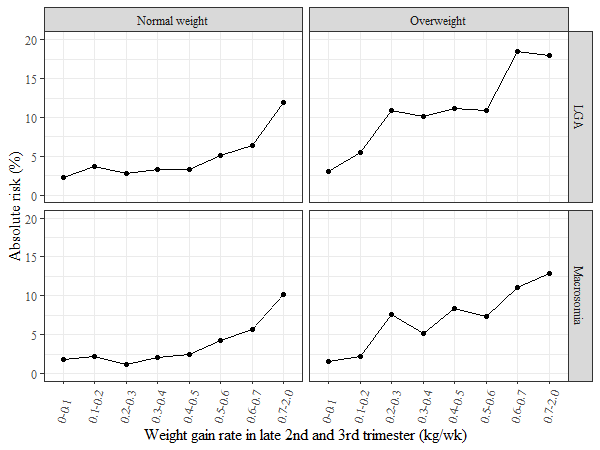

Supplement: Supplementary file 1 — Additional file 1. [file 12884_2022_4762_MOESM1_ESM.docx]
